# Germline *ERBB2*/*HER2* Coding Variants Are Associated with Increased Risk of Myeloproliferative Neoplasms

**DOI:** 10.3390/cancers13133246

**Published:** 2021-06-29

**Authors:** Evan M. Braunstein, Hang Chen, Felicia Juarez, Fanghan Yang, Lindsay Tao, Igor Makhlin, Donna M. Williams, Shruti Chaturvedi, Aparna Pallavajjala, Theodoros Karantanos, Renan Martin, Elizabeth Wohler, Nara Sobreira, Christopher D. Gocke, Alison R. Moliterno

**Affiliations:** 1 Department of Medicine, Division of Haematology, Johns Hopkins University School of Medicine, Baltimore, MD 21205, USA; h.chen@jhu.edu (H.C.); felicia.juarez18@gmail.com (F.J.); fyang28@jhu.edu (F.Y.); ltao7@jhu.edu (L.T.); dwilliams@jhmi.edu (D.M.W.); schatur3@jhmi.edu (S.C.); amoliter@jhmi.edu (A.R.M.); 2 Department of Medicine, Division of Hematology & Oncology, University of Pennsylvania, Philadelphia, PA 19104, USA; igor.makhlin@uphs.upenn.edu; 3 Department of Pathology, Johns Hopkins University School of Medicine, Baltimore, MD 21205, USA; apallav2@jhmi.edu (A.P.); cgocke1@jhmi.edu (C.D.G.); 4 Department of Medical Oncology, Johns Hopkins University School of Medicine, Baltimore, MD 21205, USA; tkarant1@jhmi.edu; 5 McKusick-Nathans Department of Genetic Medicine, Johns Hopkins University School of Medicine, Baltimore, MD 21205, USA; rmart120@jhmi.edu (R.M.); esquibb1@jhmi.edu (E.W.); nsobrei2@jhmi.edu (N.S.)

**Keywords:** familial cancer, myeloproliferative neoplasms, germline predisposition

## Abstract

**Simple Summary:**

Familial clustering of myeloproliferative neoplasms (MPN) is well known, with a 5- to 7-fold increased risk of MPN among first-degree relatives of MPN patients. However, the genetic susceptibility of this disease remains poorly understood. Exome sequencing of a familial MPN pedigree followed by a case-control analysis identified germline variants in the *HER2/ERBB2* gene that associate with the MPN phenotype. *ERBB2*/*HER2* is a novel susceptibility locus which contributes to cancer risk in combination with additional risk alleles.

**Abstract:**

Familial cases of myeloproliferative neoplasms (MPN) are relatively common, yet few inherited risk factors have been identified. Exome sequencing of a kindred with a familial cancer syndrome characterized by both MPN and melanoma produced a germline variant in the *ERBB2/HER2* gene that co-segregates with disease. To further investigate whether germline *ERBB2* variants contribute to MPN predisposition, the frequency of *ERBB2* variants was analyzed in 1604 cases that underwent evaluation for hematologic malignancy, including 236 cases of MPN. MPN cases had a higher frequency of rare germline *ERBB2* coding variants compared to non-MPN hematologic malignancies (8.9% vs. 4.1%, OR 2.4, 95% CI: 1.4 to 4.0, *p* = 0.0028) as well as cases without a blood cancer diagnosis that served as an internal control (8.9% vs. 2.7%, OR 3.5, 95% CI: 1.4 to 8.3, *p* = 0.0053). This finding was validated via comparison to an independent control cohort of 1587 cases without selection for hematologic malignancy (8.9% in MPN cases vs. 5.2% in controls, *p* = 0.040). The most frequent variant identified, *ERBB2* c.1960A > G; p.I654V, was present in MPN cases at more than twice its expected frequency. These data indicate that rare germline coding variants in *ERBB2* are associated with an increased risk for development of MPN. The *ERBB2* gene is a novel susceptibility locus which likely contributes to cancer risk in combination with additional risk alleles.

## 1. Introduction

The myeloproliferative neoplasms (MPN) are usually sporadic diseases characterized by increased JAK/STAT pathway signaling due to a somatic driver mutation in either the *JAK2*, *CALR,* or *MPL* gene. Familial cases of MPN are well described; approximately 10% of MPN cases display familial clustering, and there is a 5- to 7-fold increased risk of developing an MPN among first-degree relatives of MPN patients [1,2,3]. Similar to other familial hematologic cancers, affected families tend to display an autosomal dominant inheritance pattern with incomplete penetrance. In contrast to other myeloid malignancies, investigation of large pedigrees with familial clustering of MPN has failed to identify high-risk predisposition genes. To date, germline variants in the gene *RBBP6* were found to co-segregate with disease in three pedigrees with familial MPN, albeit with incomplete penetrance [4]. In addition, a germline duplication on chromosome 14q32 has been linked to predisposition to MPN as well as other myeloid malignancies in multiple families, though the causative gene(s) remain(s) unclear [5,6]. Recently, MPN was found to be part of the spectrum of hematologic malignancies in a large cohort with germline *DDX41* variants [7]. However, in the majority of familial MPN cases, the genetic risk factors leading to development of malignancy are unknown.

The most well-studied germline predisposition variant is a common haplotype of the *JAK2* gene, referred to as 46/1 or GGCC, which is associated with a 2- to 3-fold increased risk of acquiring a somatic JAK2 p.V617F mutation [8,9,10]. This haplotype alone does not explain familial clustering in MPN; it is equally represented in both familial and sporadic cases and is also frequently present in unaffected individuals [11]. Additional common germline single-nucleotide variants (SNVs) that are associated with increased susceptibility for MPN have been identified via genome wide association studies (GWAS). These include low-risk alleles in *TERT*, *TET2*, *SH2B2,* and other genes [12,13,14]. Interestingly, many of these same loci, particularly in the *TERT* gene, are also known to be associated with an increased risk for various solid tumor malignancies including lung, colorectal, and skin [15,16]. A recent GWAS analysis of nearly 4000 MPN cases identified 15 genes harboring potential risk variants for MPN predisposition, 3 of which (*TERT*, *ATM*, *SH2B3*) have known associations with other cancers [17].

Despite these recent advances, discovery of variants with direct influence on MPN predisposition has remained elusive. In this study, we identified a rare germline variant in the gene *ERBB2* that co-segregates with disease in a family with an autosomal dominant familial cancer syndrome characterized by both MPN and solid tumor malignancies. The *ERBB2* gene is a tyrosine kinase receptor (also known as *HER2*) that is frequently amplified or mutated in cancer [18]. Somatic mutations in *ERBB2* are relatively common in many solid tumor malignancies; however, mutations in hematologic diseases are infrequent [19]. Analysis of targeted sequencing of *ERBB2* in multiple disease and control cohorts revealed that rare germline *ERBB2* coding variants are more frequently associated with MPN compared to other hematologic malignancies as well as non-cancer controls. These data implicate germline *ERBB2* variants as potential risk factors for development of MPN.

## 2. Materials and Methods

### 2.1. Patient Recruitment

The study was approved by the Johns Hopkins University institutional review board (IRB) and complied with all required ethical standards. Written informed consent was obtained from all participants when consent waiver was not applicable. Patients either consented and were enrolled into a research registry for the purposes of genetic sequencing, or they underwent genetic sequencing as part of their clinical evaluation.

### 2.2. Whole Exome Sequencing and Variant Analysis

DNA samples were obtained via peripheral blood or saliva using the Gentra Puregene kit (Qiagen, Germantown, MD, USA). Whole exome sequencing (WES) completed by the Baylor-Hopkins Center for Mendelian Genomics as previously described [20], utilizing SureSelect HumanAllExonV5Clinical_S06588914 kit (Agilent, Savage, MD, USA)to capture ~51 Mb of CCDS exonic regions and flanking intronic regions. A total of 125 bp paired-end reads were sequenced using the HiSeq PE Cluster Kit v4 (Illumina, San Diego, CA, USA) method on the Illumina HiSeq2500. Local realignment and variant calling were performed using BWA mem 0.7.8 alignment with GATK 3.0 joint calling with HaplotypeCaller/CombineGVCF/GenotypeGVCF workflow. Variants were then filtered using the Variant Quality Score Recalibration (VQSR) method. The sequencing coverage and quality statistics for each sample are summarized in Table S5.

Analysis was performed using PhenoDB’s Variant Analysis Module [21]. An analysis that assumed an autosomal dominant mode of inheritance was run in PhenoDB for this family (affected proband, affected mother, and affected maternal aunt) to select for shared, rare (minor allele frequency (MAF) <1% in the 1000 Genomes Project, Exome Variants server (release ESP6500SI) and gnomAD databases [22,23]), and functional (missense, nonsense, frameshift, splice site, and indels) heterozygous variants present in all affected individuals.

### 2.3. Variant Confirmation

PCR of candidate genes was performed using primers listed in Table S2. PCR products were purified prior to Sanger sequencing using ExoSAP-IT (Affymetrix, Santa Clara, CA, USA). Sanger sequencing was performed by the Johns Hopkins Genetic Resources Core Facility (Baltimore, MD, USA).

### 2.4. Targeted Sequencing

DNA samples were obtained from peripheral blood or bone marrow for all cases as previously reported [24]. Targeted sequencing of the *ERBB2* gene was performed using an Illumina TruSeq Custom Amplicon (v1.5) kit (San Diego, CA, USA) designed to sequence the entire open reading frame of selected genes. Amplicon libraries were generated following the manufacturer’s protocol (Illumina). Paired-end sequencing was performed using MiSeq Reagent Kit v2 (2 × 250 bp) (Illumina, San Diego, CA, USA). Analysis was performed using Illumina BaseSpace TruSeq applications with alignment using the banded Smith–Waterman algorithm and variant calling by GATK 1.6. Sequencing coverage and quality statistics are summarized in Table S6. Germline variants were identified with variant allele frequency between 40–60%, and variants with an MAF > 0.01 in the gnomAD database v2.1.1 were removed. Heterozygous, nonsynonymous variants with a depth >40× were selected for further analysis.

### 2.5. Clinical Sequencing Analysis

All patients underwent clinical evaluation at the Johns Hopkins Hospital with diagnoses made by clinical pathologists based on World Health Organization criteria. Next-generation sequencing was performed from peripheral blood or bone marrow samples by the Molecular Diagnostics Laboratory at Johns Hopkins Genomics using a 640 gene panel as described [25]. An in-house variant caller (MDL VC 8.0) and a third-party variant caller (Haplotyper Genome Analysis TK-3.3) using the Bayesian statistical model were employed to generate a list of variants. These variants underwent further filtering utilizing in-house algorithms and annotation utilizing the COSMIC database v88, dbSNP v150, and Annovar (7042018) to assess germline variant status. Sequencing coverage for each variant call reported in this study is summarized in Table S7.

### 2.6. Statistical Analysis

Odds ratios (OR) and 95% confidence intervals (CI) were calculated by comparing the proportion of rare coding variant carriers among cases with the proportion of carriers among controls. All statistical tests were two sided and were considered significant at the alpha = 0.05 level using Pearson’s chi-squared test. Analysis was performed using SPSS. Observed/expected (o/e) ratios for individual variants were calculated using the global MAF reported in gnomAD v2.1.1 for each variant.

## 3. Results

### 3.1. Germline ERBB2 Variants in Familial MPN

**Figure 1:**
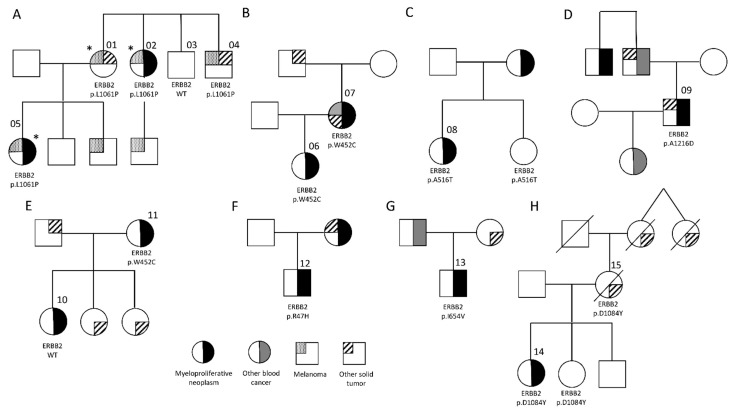
Pedigrees of families with inherited MPN. (**A**) Exome sequencing of the three family members marked with an asterisk (*) was performed in order to identify the predisposing inherited variant in this family. Sequence analysis produced a shared heterozygous variant in the gene *ERBB2*. Genotyping of two additional family members for the ERBB2 c.3182T > C; p.L1061P variant established that it co-segregates with the cancer phenotype. (**B–G**) Pedigrees of the 6 additional cases of familial MPN found to have germline *ERBB2* variants. (**H**) An MPN case with a germline ERBB2 c.3250G > T; p.D1084Y variant was noted to have a family history of breast cancer in her mother, who also carried this variant. Her sister was identified as an unaffected carrier. See Table S1 for additional pedigree information.

In order to identify novel predisposition genes in MPN, we investigated a family with a highly penetrant, autosomal dominant familial cancer syndrome (Figure 1A, Table S1). The proband was diagnosed with polycythemia vera (PV) at age 38, with a JAK2 p.V617F variant allele frequency (VAF) of 47%. Subsequent personal cancer history included melanoma (diagnosed one year later) and renal carcinoma (diagnosed five years later). Family history was significant for ovarian cancer and melanoma in her mother, essential thrombocytosis (ET) and melanoma in her maternal aunt, bladder cancer and melanoma in her maternal uncle, and melanoma in her brother. Exome sequencing of three family members (* in Figure 1A) was performed in order to identify the predisposing inherited variant in this family. Analysis directed at variants following an autosomal dominant mode of inheritance produced eight shared SNVs (Table S2). Genotyping of two additional family members for these eight candidates identified SNVs in three candidate genes that co-segregated with the cancer phenotype in this family—*ERBB2*, *MCM4*, and *DOCK5*.

Germline variants of *ERBB2* have been associated with hereditary lung carcinoma and identified as a potential risk factor for breast cancer and melanoma [26,27,28,29]. However, an association with hematologic malignancies has not been reported. We previously identified a rare *ERBB3* germline variant that co-segregated with disease in a pedigree with familial erythroid myelodysplastic syndrome/acute myeloid leukemia (MDS/AML), implicating this gene family in predisposition to blood cancers [30]. The family described here (Figure 1A) harbored an *ERBB2* c.3182T > C; p.L1061P variant that has been reported as a rare germline variant most commonly seen in individuals of Ashkenazi Jewish decent, consistent with the ethnicity of the proband. In addition, it is absent from the Genomic Data Commons (GDC), which contains somatic mutation data from thousands of cancer cases [31]. Thus, we further investigated the *ERBB2* gene as a candidate for germline predisposition to hematologic malignancy.

We initially investigated the hypothesis that germline *ERBB2* variants predispose to familial MPN by performing targeted sequencing in an additional 55 unrelated cases of familial MPN. This was defined as a diagnosis of MPN in the index case, as well as MPN or other myeloid malignancies such as MDS or AML in a first- or second-degree relative. Targeted sequencing of the coding regions of *ERBB2* was performed, with isolation of variants with a global minor allele frequency (MAF) < 0.01. In this cohort, an additional 6 cases with a germline *ERBB2* variant were identified (Figure 1), giving a total of 7 out of 56 (12.5%) unrelated cases of familial MPN harboring *ERBB2* coding variants. No correlation between MPN subtype or driver mutation was observed (Table S1). Genotyping of additional family members was possible for two pedigrees in which the probands harbored the ERBB2 p.W452C variant (Figure 1B,E). Despite its relatively common frequency in the African population (MAF = 0.049), this variant is predicted to be potentially damaging via PolyPhen-2 [32]. However, it co-segregated with the MPN phenotype in only one of the two families, suggesting that this variant is less likely to contribute to MPN predisposition.

### 3.2. Frequency of ERBB2 Variants in Hematologic Malignancies

The analysis of our familial MPN cohort showed that rare *ERBB2* germline variants were prevalent in 12.5%, although none of the variants harbored a lesion identical to that of our index family. Analysis of inherited predisposition lesions is confounded by incomplete penetrance and variable expressivity of phenotypes in cancer predisposition syndromes. Previous studies have suggested that genetic predisposition in MPN may be due to an increase in somatic mutability and a rise in overall carcinogenesis [2,11]. Supporting this hypothesis, the cancer histories of the individuals in Figure 1 are not restricted to MPN. To investigate the hypothesis that rare germline *ERBB2* variants predispose to multiple types of hematologic neoplasms, targeted sequencing of *ERBB2* was analyzed in a cohort of 1604 consecutive individuals who underwent clinical evaluation for hematologic malignancy over a 5-year period from 2015 to 2020. This cohort comprised myeloid malignancies such as MDS, AML, MPN, chronic myelomonocytic leukemia (CMML), chronic myeloid leukemia (CML), and aplastic anemia (AA), as well as a mixture of lymphoid malignancies. In addition, 256 individuals in this cohort who underwent evaluation showed no hematologic malignancy and were used as controls.

To increase the likelihood of isolating potentially pathogenic variants, SNVs with an MAF > 0.01 in any ethnic subgroup in the gnomAD database were excluded. Analysis of the entire cohort of hematologic malignancies revealed 67 of 1348 cases (5.0%) harboring a rare germline *ERBB2* coding variant, compared to 7 of 256 (2.7%) in the control cohort (Table S3). Similar results were obtained using a more restrictive MAF threshold of 0.005, with 1.9% (26 of 1348) in the malignant cohort compared to 1.2% in the controls (3 of 256). While both analyses revealed a trend toward an increased frequency of germline *ERRB2* variants in hematological malignancies, statistical significance was not met (*p* = 0.11 and *p* = 0.41 for MAF < 0.01 and 0.005, respectively). We next investigated the frequency of *ERBB2* variants stratified by diagnosis. Interestingly, patients with MPN harbored rare germline *ERBB2* coding variants at a significantly increased frequency compared to controls (Table 1). This was true for variants with an MAF < 0.01 (8.9% vs. 2.7%, *p* = 0.0053) and an MAF < 0.005 (4.7% vs. 1.2%, *p* = 0.031). Germline *ERBB2* variants were also more frequent in MPN cases compared to those with MDS/AML and when compared to all non-MPN hematologic malignancies. In this analysis, additional family members were available for one case of PV with a family history of breast cancer in both her mother and grandmother (Figure 1H, Table S1). This individual harbored an *ERBB2* c.3250G > T; p.D1084Y variant, reported as a rare germline variant in Europeans and absent from tumor samples in the GDC database. This variant was identified in the proband’s mother as well as an unaffected sister, indicating that it co-segregates with disease in this family with incomplete penetrance.

These data indicate that rare germline *ERBB2* coding variants are more frequent in individuals with MPN than in those with other hematologic malignancies and those without a cancer diagnosis. No association with MPN disease subtype or driver mutation was observed. To validate these findings, we utilized sequencing data from 1587 unrelated individuals studied via the Baylor-Hopkins Center for Mendelian Genomics (BHCMG). Individuals enrolled in the BHCMG to investigate an inherited cancer disorder were excluded, and *ERBB2* coding variants with an MAF < 0.01 in any population subgroup were isolated (Table S4). In this analysis, *ERBB2* variants were more frequent in cases with MPN compared to the control cohort (8.9% vs. 5.2%, *p* = 0.040), confirming the small but significant MPN risk associated with variants in this gene (Table 1). The same analysis using variants with an MAF < 0.005 produced a non-statistically significant trend toward an increased frequency of *ERBB2* variants in MPN (4.7% vs. 3.0%, *p* = 0.169).

### 3.3. ERBB2 Variants Overrepresented in MPN

Analysis of specific variants was performed using the combined MPN cohort of 292 cases. This cohort was compared to 1112 non-MPN blood cancers and 1587 unselected controls (validation cohort). The most frequent germline variant identified in all cohorts was *ERBB2* c.1960A > G; p.I654V (Table 2). This heterozygous variant occurred in 14% (1/7) of the familial MPN cohort (Figure 1G), 2.05% of the MPN cohort, 1.53% of the non-MPN cancer cohort, and 1.07% of the control cohort. Based on its known population frequency, p.I654V was present in the MPN cohort twice as often as expected (observed/expected (o/e) ratio of 2.13) compared to an o/e ratio of 1.59 in the non-MPN cancer cohort and 1.11 in the control cohort (Table 2). Interestingly, p.I654V has been previously correlated with an increased risk of both breast cancer and melanoma [26,27]. Supporting this, a family history of breast cancer was present in the individual harboring a p.I654V variant in Figure 1G. Other variants that were identified more than once in cancer cases are displayed in Table 2, and an o/e ratio was calculated for each variant. The p.E930D variant was most frequent in the MPN cohort with an o/e ratio of 6.67, compared to 1.75 in the non-MPN cancer cohort and 0.613 in the control cohort. A third variant, p.P489L, was also more frequent in the MPN cohort with an o/e ratio of 2.36. Conversely, the o/e ratio for each of these variants is close to the expected value of 1 in the control cohort. One outlier, the extremely rare variant p.I961T, was identified in two cases of the non-MPN cancer cohort and was absent from both MPN and control cases.

## 4. Discussion

Familial clustering in MPN is well described and relatively common; however, few highly penetrant predisposing germline variants have been identified to date. This contrasts with other myeloid hematologic malignancies, where a number of high-risk susceptibility genes have been discovered [33]. One explanation for this is that high-risk susceptibility predisposition genes are quite rare in MPN and are responsible for an extremely small number of cases. Further, it suggests that familial clustering in MPN may be explained via the co-inheritance of multiple, independent, dominant variants, each with a weak to moderate effect on disease susceptibility. Evidence for an oligogenic model of inherited susceptibility has been reported in breast cancer as well as other cancer types [34,35] and is consistent with the rare-variant hypothesis for genetic predisposition [36].

We have identified a rare heterozygous germline *ERBB2* missense variant that co-segregates with disease in a familial cancer syndrome manifesting as MPN, melanoma, and other solid tumors. In addition, we show that germline *ERBB2* variants are more frequently identified in individuals with an MPN phenotype compared to those with other hematologic malignancies and non-cancer controls. Together, these data implicate rare *ERBB2* variants in the predisposition to MPN and possibly other cancer syndromes. We propose that germline *ERBB2* variants belong to a class of susceptibility variant that falls in between very rare variants with high disease penetrance (i.e., *TP53* variants in Li–Fraumeni syndrome, or *BRCA1* variants in breast and ovarian cancer), and common but low-risk susceptibility alleles detected via GWAS. In contrast to common susceptibility alleles, variants such as those identified in this study are thought to exert a direct (albeit small) functional effect that contributes to the disease phenotype in the form of a mild dominant or dominant negative effect [37]. Though the effect of a single variant is small, the combination of multiple risk alleles, including more common ones such as the *JAK2* 46/1 haplotype, produces a significant disease risk.

**Figure 2:**
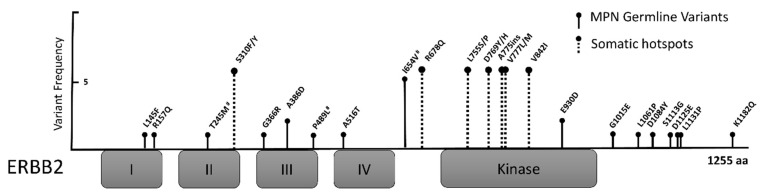
Germline ERBB2 variants identified via target sequencing. Location of germline variants in the ERBB2 protein identified in cases with a myeloproliferative neoplasm. The amplitude of the “stem” for each germline variant corresponds to its frequency in the cohort. Variants are found throughout the protein, but they spare somatic hotspot regions (dashed lines) including p.S310 and the kinase domain. Extracellular domains are labelled I–IV, and the kinase domain is indicated. # denotes germline variants that have been previously reported in cancer predisposition. aa, amino acids.

The *ERBB2* gene (also called *HER2*) is a well-described oncogene that is commonly amplified or overexpressed in human cancers [38]. Somatic mutations in *ERBB2* are reported in many solid tumor malignancies, most commonly in bladder, uterine, colon, and gastric cancers; mutations in hematologic diseases are rare but have been reported [39,40]. The ERBB2 somatic mutations found in cancer samples are usually located in the kinase domain (KD) or hotspots in the extracellular domains (ECD) and have been shown to activate ERBB2 signaling [39,41,42]. Interestingly, the germline variants identified in this study are located throughout the ERBB2 protein, but they notably spare the KD and ECD hotspot regions (Figure 2), similar to germline *ERBB2* variants reported in breast, lung, melanoma, and pediatric cancers [26,27,28,43,44]. This is consistent with a model in which germline variants produce weak protein activation that is unable produce a cancer phenotype alone, but when combined with multiple additional low-risk variants, the effect becomes significant. Elucidation of the contribution of germline *ERBB2* variants to MPN and possibly other cancer types will require functional studies. It is possible, however, that germline variants induce a chronic, low-level of ERBB signaling and subsequent activation of the JAK/STAT, PI3K/AKT, or MEK/ERK pathways, with convergence on common downstream targets. Alternatively, *ERBB2* may exert its predisposition to MPN acquisition by pathways that are not intrinsic or specific to hematopoietic cell signaling or development, such as genomic instability, inflammation, telomere biology, or other pathways implicated in cancer susceptibility [17,45,46,47].

## 5. Conclusions

In summary, germline coding variants in *ERBB2* are more common among individuals with MPN than individuals without cancer. In particular, ERBB2 p.I654V is associated with an approximately two-fold increase in risk of MPN compared to controls. This and other germline *ERBB2* variants convey a small but significant increased risk of disease and likely contribute to familial clustering in cancer syndromes.

## Figures and Tables

**Table 1:** Evaluation of germline ERBB2 variants identified and cancer cases and controls.

**Diagnosis**	**N Cases**	**ERBB2 MAF < 0.01**	**ERBB2 MAF < 0.005**
MPN	236	21 (8.9%)	11 (4.7%)
CMML	92	6 (6.5%)	2 (2.2%)
MDS/AML	771	30 (3.9%)	9 (1.2%)
AA	61	3 (4.9%)	2 (3.3%)
CML	27	1 (3.7%)	0
Lymphoid	161	6 (3.7%)	1 (0.06)
Control (Non-Blood Cancer)	256	7 (2.7%)	3 (1.2%)
All Blood Cancer	1348	67 (5.0%)	26 (1.9%)
Non-MPN Blood Cancer	1112	46 (4.1%)	14 (1.3%)
Validation Control Cohort	1587	83 (5.2%)	47 (3.0%)
**Comparison**	**Odds ratio (95% confidence interval), *p* value**		
MPN vs. Control		3.5 (1.4 to 8.3), *p* = 0.0053	4.1 (1.1 to 13.5), *p* = 0.0313
MPN vs. MDS/AML		2.4 (1.4 to 4.0), *p* = 0.0028	4.1 (1.7 to 10.1) *p* = 0.0018
MPN vs. Non-MPN Blood Cancer		2.3 (1.3 to 3.9) *p* = 0.0028	3.8 (1.7 to 8.6) *p* = 0.0010
MPN vs. Validation Control		1.7 (1.0 to 2.8) *p* = 0.0400	1.6 (0.8 to 3.1) *p* = 0.1698

AML, acute myeloid leukemia; AA, aplastic anemia; CMML, chronic myelomonocytic leukemia; MAF, minor allele frequency; MDS, myelodysplastic syndrome; MPN, myeloproliferative neoplasm.

**Table 2:** ERBB2 variants identified in multiple cancer cases.

	MPN Cohort (*n* = 292)			Non-MPN Cancer Cohort (*n* = 1112)			Validation Control Cohort (*n* = 1587)		
Variant	Observed (%)	Expected #	O/E Ratio	Observed (%)	Expected #	O/E Ratio	Observed (%)	Expected #	O/E Ratio
I654V	6 (2.05)	2.81	2.13	17 (1.53)	10.72	1.59	17 (1.07)	15.29	1.11
A386D	2 (0.68)	2.12	0.94	8 (0.72)	8.09	0.99	13 (0.82)	11.55	1.13
E930D	2 (0.68)	0.30	6.67	2 (0.18)	1.14	1.75	1 (0.06)	1.63	0.61
P489L	1 (0.34)	0.42	2.36	3 (0.27)	1.61	1.86	2 (0.13)	2.30	0.87
R1161Q	0	0.44	NA	3 (0.27)	1.69	1.78	2 (0.13)	2.41	0.83
R143Q	0	0.28	NA	2 (0.18)	1.10	1.89	0	1.51	NA
I961T	0	0.004	NA	2 (0.18)	0.02	113	0	0.03	NA

# based on global MAF.

## Data Availability

The WES data generated in this study will be deposited in AnVIL (https://anvilproject.org) by the BHCMG per the policy of the Center for Mendelian Genomics. Targeted NGS sequencing data and other data that support the findings of this study are available from the corresponding author upon request.

## Supplementary Materials

The following are available online at https://www.mdpi.com/article/10.3390/cancers13133246/s1, Table S1: Familial MPN cases harboring a germline ERBB2 variant, Table S2: Candidate variants from exome sequencing of pedigree A, Table S3: Germline ERBB2 variants identified in hematologic malignancy cases and controls, Table S4: Germline ERBB2 variants identified in validation control cohort, Table S5: Whole genome sequencing coverage statistics, Table S6: Amplicon sequencing statistics for familial MPN cohort, Table S7: Sequencing statistics for the 75 variant calls in Table S3.
